# Hospitalizations with post-traumatic stress disorder in France between 2013 and 2022: a nationwide retrospective study

**DOI:** 10.1192/j.eurpsy.2024.1810

**Published:** 2025-01-15

**Authors:** Alice Demesmaeker, Florian Dufrenois, Chloé Saint-Dizier, Guillaume Vaiva, Antoine Lamer, Mathilde Horn, Fabien D’Hondt

**Affiliations:** 1 Univ. Lille, Inserm, CHU Lille, U1172 - LilNCog - Lille Neuroscience & Cognition, Lille, France; 2 Centre national de ressources et de résilience (Cn2r), Lille-Paris, France; 3 Fédération Régionale de Recherche en Santé Mentale et Psychiatrie des Hauts-de-France – F2RSM Psy, Saint-André-Lez-Lille, France; 4 Univ. Lille, CHU Lille, ULR 2694 – METRICS: Évaluation des Technologies de Santé et des Pratiques Médicales, Lille, France

**Keywords:** Posttraumatic stress disorder, hospitalization, comorbid disorders, epidemiology

## Abstract

**Introduction:**

The lifetime prevalence of PTSD ranges from 6 to 20% and is often associated with comorbid disorders. Despite the significant impact of PTSD, specific data on healthcare utilization related to PTSD remain limited. This study aims to characterize PTSD-related hospitalizations in France over the past decade.

**Methods:**

This nationwide longitudinal retrospective study analyzed PTSD-related hospitalizations in France from 2013 to 2022 using the French National Hospitals Database. Data included discharge records from general and psychiatric hospitals, detailing demographics, admission/discharge dates, ICD-10 diagnoses, and hospitalization specifics.

**Results:**

Between 2013 and 2022, 69,108 patients underwent 125,349 hospitalizations with a PTSD diagnosis (0.4% of all inpatient cases) in France. Psychiatric facilities accounted for 74,988 hospitalizations (1% of all psychiatric inpatient cases in France), while general hospitals recorded 50,361 hospitalizations (0.02% of all non-psychiatric inpatient cases). The percentage of inpatients diagnosed with PTSD increased from 0.68 to 2.22% in psychiatric facilities and from 0.02 to 0.04% in general hospitals over the study period. Females were younger in both settings and had longer stays compared to males in psychiatric facilities. Over time, there was a decrease in median age and an increase in part-time hospitalizations in psychiatric facilities. Mood disorders, stress-related disorders, and substance use disorders were prevalent comorbidities in both settings.

**Conclusions:**

This study highlights a rise in PTSD-related hospitalizations in France, particularly in psychiatric facilities and after 2019, with high rates among women and an increase in hospitalization of younger individuals. These findings highlight the necessity for improved care strategies tailored to the increasing number of younger patients with PTSD.

## Introduction

Approximately 70% of adults worldwide will experience a traumatic event, such as death, threatened death, actual or threatened serious injury, or sexual violence. Among those exposed to trauma, it has been estimated that 12% will subsequently develop post-traumatic stress disorder (PTSD) [1–3]. PTSD is a psychiatric disorder characterized by persistent intrusion symptoms, avoidance of trauma-related stimuli, negative alterations in mood and cognition, and arousal symptoms [4]. With a lifetime prevalence of 13.0 to 20.4% for women and of 6.2 to 8.2% for men, PTSD is not only common but also carries a heavy burden [5, 6] due to its invalidating symptoms and its frequent co-occurrence with both psychiatric and non-psychiatric conditions, which contribute to increased healthcare utilization and premature mortality [7, 8].

Around 90% of individuals with PTSD have at least one co-occurring mental health condition [9], such as major depression, substance use disorder, anxiety disorders, and suicidal behavior [6]. Additionally, those with PTSD are twice as likely to have a non-psychiatric condition, including chronic pain, inflammation, cardiometabolic disorders, cancer, and dementia, compared to those without PTSD [7, 10–15]. These comorbidities can arise concurrently with PTSD due to shared causal factors or as a consequence of the disorder itself [11]. Consequently, individuals with PTSD exhibit more frequent and longer hospitalizations in general hospitals [16–18].

Despite the high prevalence and significant impact of PTSD, there is limited literature specifically addressing related hospitalizations [19–21]. This gap is problematic because it means that the healthcare burden, particularly the patterns and trends of hospitalizations, is not well understood. The only nationwide existing study, conducted between 2002 and 2011 in the United States, highlights a rising trend in PTSD-related hospitalizations, particularly among women aged 20–44 [19]. Most PTSD-related hospitalizations in this study were associated with a primary diagnosis of a mental disorder, followed by circulatory system diseases in men and alcohol or drug-induced diseases in women [19]. Additionally, this study, along with other limited literature, suggests that individuals with PTSD are more frequently female and younger adults [16, 19, 22]. Importantly, the recent coronavirus disease 2019 (COVID-19) pandemic is likely to impact these previous findings, as converging data suggest that PTSD rates have increased due to pandemic-related stressors [23–26]. Understanding how the pandemic may have influenced trends in hospitalizations is crucial, as it could reveal new patterns and demands on the healthcare system that were not apparent in earlier studies.

To provide updated data, we analyzed data from the French National Hospitals Database, offering a comprehensive overview of PTSD-related hospitalizations from 2013 to 2022. Our research aims to estimate the number of hospitalizations with PTSD, determine inpatient prevalence in general and psychiatric facilities, and explore socio-demographic characteristics, hospital features, and comorbidities associated with PTSD. We hypothesize that the number of hospitalizations with a primary or secondary diagnosis of PTSD will increase over time between 2013 and 2022, with a notable rise after 2019, due to the impact of the COVID-19 pandemic. We expect hospitalization rates to be higher in psychiatric facilities compared to general hospitals. Additionally, we hypothesize that females and younger adults will be more frequently hospitalized and that PTSD will co-occur with a high number of psychiatric comorbidities, such as depression, alcohol use disorder, and anxiety disorders.

## Method

### Study design

We conducted a nationwide longitudinal retrospective study, including all discharges between 2013 and 2022 from general and psychiatric hospitals in France with PTSD diagnosis.

### Data collection

We collected data from the French National Hospitals Database (PMSI, for *Programme de médicalisation des systèmes d’information*, in French). This database includes standardized discharge reports for all inpatient stays in private and public hospitals in France. The data encompasses sociodemographic information (such as sex and age), dates of admission and discharge, type of facility, and primary and secondary diagnoses (i.e. main cause for admission and comorbidities). This French National Hospitals Database records the primary diagnosis (i.e. the main reason for admission) and the secondary diagnoses (i.e. comorbidities) reported by physicians during hospitalization according to the French version of the International Statistical Classification of Diseases and Related Health Problems, 10th Revision (ICD-10) [27]. PTSD is identified by the ICD-10 code F43.1.

### Population

All hospitalizations with a primary or secondary diagnosis of PTSD between 2013 and 2022 were included in the analysis. No exclusion criteria were applied. Patients treated in outpatient settings are not included in this database.

### Ethical approval

Ethical approval was not required for this study as we accessed an anonymous administrative database. Additionally, the National French Public Health Agency (*Santé Publique France*) permits full access to national hospital discharge databases, including the PMSI, which is commonly utilized for research. The authors affirm that all procedures conducted in this study adhere to the ethical standards set by the pertinent national and institutional committees on human experimentation, as well as the principles outlined in the Helsinki Declaration of 1975, as revised in 2008.

### Variables

We utilized multiple variables from the PMSI database, including age and sex, admission and discharge dates, primary and secondary diagnoses, facility type (general or psychiatric hospital, private or public facility), and legal status of psychiatric hospitalization (voluntary or compulsory care). Certain variables, such as part-time or full-time hospitalization, voluntary or compulsory care, and whether the hospitalization occurred in a public or private hospital, were only available for psychiatric hospitalizations in this database. Hospital stays were divided into full-time and part-time hospitalizations, with full-time hospitalizations defined as those containing at least one period of full-time hospitalization during the hospital stay. Hospital stays were considered compulsory if they contained at least one episode of care without consent, which in France is only permitted in psychiatric hospitals in accordance with national legal regulations. Comorbidities were defined by all ICD-10 codes other than F43.1, regardless of whether they were used as the primary or secondary diagnostic.

### Statistical analyses

We described all quantitative variables with mean and standard deviation or median and 1st and 3rd quartiles (Q1–Q3). Qualitative variables were described using absolute numbers and percentages. Initially, for both types of hospitalization, we counted the number of patients and hospitalizations and calculated the mean number of hospitalizations per patient. We then evaluated age, sex, hospital stay length, and the number of yearly hospitalizations. For hospitalizations from psychiatric hospitals, we also described part or full-time hospitalization, type of facility (private or public), and compulsory or voluntary care. For hospitalizations in general hospitals, we specifically examined the original input of care. Additionally, we compared age, hospital stay length, and hospital-type specific factors between sexes and between 2013 and 2022 using Wilcoxon and Chi-2 tests. *p*-Values under 0.05 were considered significant. Finally, we examined the most frequent comorbidities. All data management, statistical analyses, tables, and figures were realized using SAS 8.3 and R 4.1.2.

## Results

### Hospitalizations with a diagnosis of PTSD

From 2013 to 2022, 69,108 patients underwent a total of 125,349 hospitalizations with a primary or secondary diagnosis of PTSD in France (Table 1). Among them, 34,436 patients underwent 74,988 hospitalizations in psychiatric facilities, and 37,104 patients underwent 50,361 hospitalizations in general hospitals. Of these patients, 2,466 were hospitalized in both types of facilities. Hospitalizations for PTSD represent 0.39% of all hospitalizations in France, 1.01% of hospitalizations in psychiatric facilities, and 0.02% of hospitalizations in general hospitals.

**Table 1.** Characteristics of hospitalizations with PTSD in France

	Overall (*N* = 125,349)	Psychiatric facilities (*N* = 74,988)	General hospital wards (*N* = 50,361)
Number of patients	69,108	34,436	37,104
Age			
Median [Q1, Q3]	37 [23, 51]	35 [23, 47]	42 [24, 63]
Sex, n (%)			
Female	74,663 (59.6)	43,357 (57.8)	31,306 (62.2)
Male	50,686 (40.4)	31,631 (42.2)	19,055 (37.8)
Lengths of stay (median, [Q1, Q3])			
All types	7 [2, 17]		4.00 [1, 10]
Full-time		13.00 [5, 29]	
Part-time		1.50 [1, 10]	
Type of hospitalization, n (%)			
Full-time		55,903 (74.5)	
Part-time		19,085 (25.5)	
Type of facility, n (%)			
Private		30,226 (40.3)	
Public		44,762 (59.7)	
Type of care, n (%)			
Voluntary		71,161 (94.9)	
Compulsory		3827 (5.1)	

N = Number of hospitalizations; Q1 = First quartile; Q3 = Third quartile.

**Figure 1.**
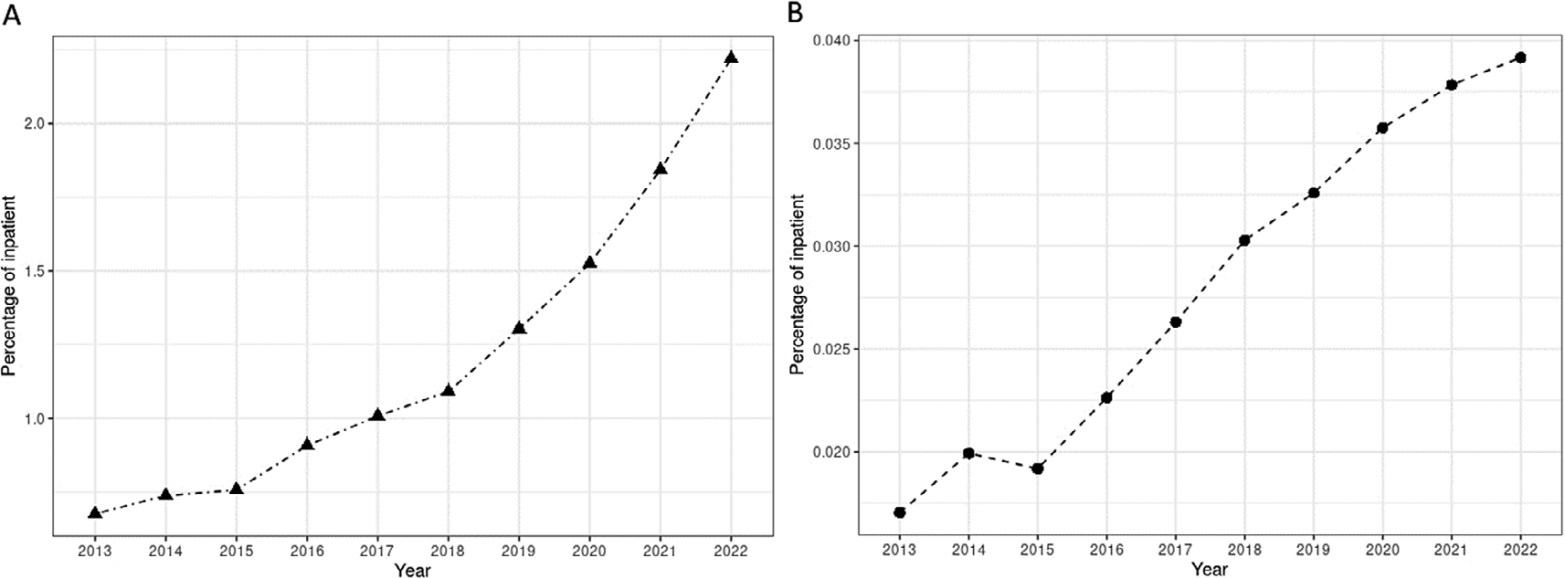
Trends in the percentage of inpatients diagnosed with PTSD from 2013 to 2022 in psychiatric facilities (A) and general hospitals (B) in France.

During the study period, the percentage of inpatients with a PTSD diagnosis in psychiatric facilities increased from 0.68 to 2.22% (see Figure 1). The prevalence of the disorder appears to increase linearly until 2019, followed by a sharper rise. In general hospitals, the percentage of inpatients with a PTSD diagnosis also increased between 2013 and 2022, from 0.02 to 0.04%. The prevalence of the disorder increased almost linearly during this period.

### Hospitalizations in psychiatric hospitals

Among those hospitalized in psychiatric facilities, 57.8% were females with a median age of 35 years (Q1–Q3: 23–47). Most hospitalizations were full-time (74.5%) and occurred in public facilities (59.7%). The median hospital stay duration for full-time hospitalizations was 13 days (Q1–Q3: 5–29), while the median number of days of presence for part-time hospitalizations was 1.5 days (Q1–Q3: 1–10). Nearly all patients were hospitalized under voluntary care (94.9%). The mean number of hospitalizations per patient was 1.96 (SD: 2.96), with more than one-third of the patients (37.3%) having at least two hospitalizations.

### Hospitalizations in general hospitals

In general hospitals, the median age at admission was 42 years (*Q*1–*Q*3: 24–63), with most patients being female (62.2%). The median hospital stay length was 4 days (*Q*1–*Q*3:1–10). Patients had on average 1.36 hospitalization (SD = 3.6), with 5,272 (13.9%) having at least two hospitalizations.

### Comparison by sex

**Figure 2.**
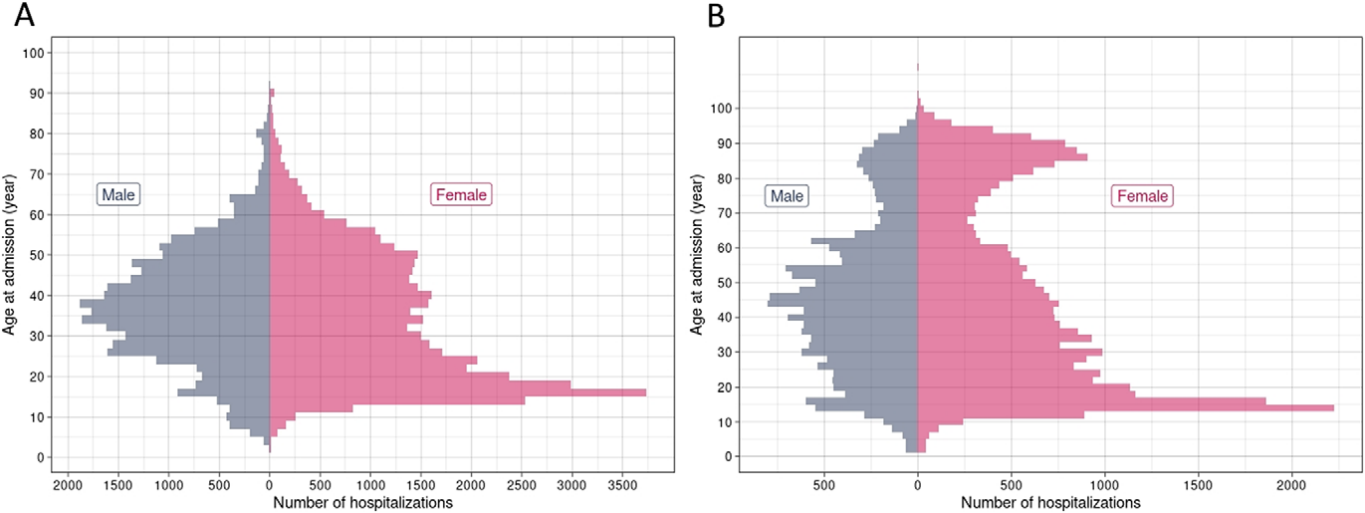
Number of hospitalizations by age at admission and sex in psychiatric facilities (A) and general hospitals (B).

When comparing psychiatric hospitalizations by sex, female patients were younger (median age: 32 years for females and 37 years for males, *p*-value < 0.01) (see Table 2 and Figure 2). Females were more frequently in full-time hospitalization (*p*-value < 0.01) and in private facilities (52.4% for females and 29.2% for males, *p*-value < 0.01). Their hospital stay tended to be longer, both full-time (median stay: 16 days for females and 11 days for males, *p*-value < 0.01) and part-time (median stay: 5 days for females and 1 day for males, *p*-value < 0.01). In general hospital wards, female patients were also younger (median age: 39 years for females and 45 years for males, *p*-value < 0.01), but tended to stay as long as males (median stay: 4 days for females and 4 days for males, *p*-value = 0.1).

**Table 2.** Comparison of hospitalizations in psychiatric and general hospital wards by sex

	Psychiatric facilities (*N* = 74,988)			General hospital wards (*N* = 50,361)		
	Female	Male	*p*-Value	Female	Male	*p*-Value
Number of hospitalizations (%)	43,357 (57.8)	31,631 (42.2)		31,306 (62.2)	19,055 (37.8)	
Age, median [Q1, Q3]	31 [20, 46]	37 [28, 47]	<0.01^a^	39 [22, 67]	45 [30, 61]	<0.01^a^
Lengths of stay, median [Q1, Q3]						
All types				4 [1, 10]	4 [1, 11]	0.1^a^
Full-time	14 [5, 33]	11 [4, 25]	<0.01^a^			
Part-time	3.5 [1, 14]	1 [1, 5]	<0.01^a^			
Type of hospitalization, *n* (%)						
Full-time	33274 (76.7)	22,629 (71.5)	<0.01^b^			
Part-time	10,083 (23.3)	9002 (28.5)				
Type of facility, *n* (%)						
Private	21,735 (50.1)	8491 (26.8)	<0.01^b^			
Public	21,622 (49.9)	23,140 (73.2)				
Type of care, *n* (%)						
Voluntary	41,397 (95.5)	29,764 (94.1)	<0.01^b^			
Compulsory	1960 (4.5)	1867 (5.9)				

N = Number of hospitalizations; Q1 = First quartile; Q3 = Third quartile.

a Results of Wilcoxon tests.

b results of Chi-2 tests.

### Evolution between 2013 and 2022

From 2013 to 2022, in psychiatric units, patients were more often in part-time hospitalization (20.8% in 2013 and 33.8% in 2022, *p*-value < 0.01) and for a shorter duration in 2022 (median number of days of presence in part-time hospitalization: 4 days in 2013 and 2 days in 2022, *p*-value < 0.01) (see Table 3). The prevalence of females tends to decrease over time, from 64.4% in 2013 to 62.2% in 2022 (*p* = 0.01). There were no differences for other variables (i.e., hospital length for full-time hospitalization, hospitalization in private or public facilities, and compulsory or voluntary care, *p* > 0.05). In general hospitals, patients were younger at admission in 2022 than in 2013, going from 47 to 39 years old (*p*-value<0.01). There were more female patients in 2022 than in 2013 (62.1% in 2013 and 65.6% in 2022, *p*-value < 0.01).

**Table 3.** Evolution between 2013 and 2022 in psychiatric and general hospital wards

	Psychiatric facilities (*N* = 74,988)			General hospital wards (*N* = 50,361)		
	2013	2022	*p*-Value	2013	2022	*p*-Value
Number of hospitalizations	3,871	13,875		2,923	7,317	
Inpatient prevalence, %	0.68	2.22		0.017	0.039	
Age, median [Q1, Q3]	36 [25, 48]	32 [20, 45]	<0.01^a^	47 [28, 69]	39 [22, 58]	<0.01^a^
Sex, *n* (%)						
Female	2493 (64.4)	8634 (62.2)	0.01^b^	1815 (62.1)	4814 (65.8)	<0.01^b^
Male	1378 (35.6)	5241 (37.8)		1108 (37.9)	2503 (34.2)	
Lengths of stay, median [Q1, Q3]						
All types				4 [1, 9]	4 [0, 10]	0.01^a^
Full-time	14 [4, 30]	14 [5, 30]	0.16^a^			
Part-time	2.5 [1, 15]	1.5 [1, 8.5]	<0.01^a^			
Type of hospitalization, *n* (%)						
Full-time	3146 (81.3)	9510 (68.5)	<0.01^b^			
Part-time	725 (18.7)	4365 (31.5)				
Type of facility, *n* (%)						
Private	1621 (41.9)	5766 (41.6)	0.74^b^			
Public	2250 (58.1)	8109 (58.4)				
Type of care, *n* (%)						
Voluntary	3689 (95.3)	13,227 (95.3)	0.97^b^			
Compulsory	182 (4.7)	648 (4.7)				

N = Number of hospitalizations; Q1 = First quartile; Q3 = Third quartile.

a Results of Wilcoxon tests.

b results of Chi-2 tests.

### Comorbidities associated with PTSD

The most frequent comorbidities in psychiatric hospitals were mood disorders (21,886, 29.2%), stress-related disorders (8,429, 11.2%), mental and behavioral disorders due to use of alcohol (7,750, 10.3%), disorders of adult personality and behavior (7,226, 9.6%), and problems related to economic and social circumstances (2,236, 3%) (Table 4). Among mood disorders, the most frequent diagnoses were major depressive disorder (15,006, 42.6%), recurrent depressive disorder (6,339, 18%), and bipolar disorder (2,608, 7.4%).

**Table 4.** Frequent comorbid disorders in PTSD-related hospitalizations

Comorbid disorders, *n* (%)	Psychiatric facilities (*N* = 74,988)	General hospital wards (*N* = 50,361)
No comorbid disorder	20,461 (27.3)	1672 (3.3)
Substance use disorders (F10–F19)	7750 (10.3)	3366 (6.7)
Schizophrenia and related disorders (F20–F29)	2407 (3.2)	217 (0.4)
Mood disorders (F30–F39)	21,886 (29.2)	3226 (6.4)
Stress-related disorders (F40–F49)	8429 (11.2)	3166 (6.3)
Behavioral syndromes (F50–F59)	3270 (4.4)	1036 (2.1)
Personality disorders (F60–F69)	7226 (9.6)	771 (1.5)
At least one non-psychiatric comorbid disorder	13,865 (18.5)	38,889 (77.2)
Problems related to economic and social circumstances (Z50–Z59)	2236 (3)	4777 (9.5)
Symptoms and signs involving the digestive system and abdomen (R50–R59)	–	2995 (5.9)

N = Number of hospitalizations.

In general hospitals, the most frequent comorbidities were problems related to economic and social circumstances (4,777, 9.5%), mental and behavioral disorders due to use of alcohol (3,366, 6.7%), mood disorders (3,226, 6.4%), stress-related disorders (3,166, 6.3%), and symptoms and signs involving the digestive system and abdomen (2,995, 5.9%).

## Discussion

We conducted a nationwide retrospective analysis over 10 years (2013–2022), focusing on hospitalizations with a primary or secondary diagnosis of PTSD in France. During this period, approximately 69,108 patients underwent a total of 125,349 hospitalizations, with the majority (59.8%) occurring in psychiatric facilities. Female patients tended to be younger and had longer hospital stays compared to males in psychiatric facilities. Additionally, we observed an increase in the number of hospitalizations with PTSD over the study period, accompanied by a significant decrease in the average age of patients at the time of hospitalization.

During the study period, PTSD-related hospitalizations in France represented 0.4% of the inpatient prevalence in the country. While the global prevalence of PTSD in the general population was estimated at 3.9% [2], we observed a lower prevalence among hospitalized individuals. This lower prevalence in hospitalizations was expected, as PTSD management primarily relies on outpatient psychotherapy [27], and hospitalizations typically reflect the most severe cases. However, previous literature suggests that PTSD could be underdiagnosed in primary and secondary care settings [28, 29]. The low prevalence of hospitalized patients with PTSD in French databases could therefore be explained by either a low prevalence of PTSD-related hospitalizations or by underdiagnosis during hospital stays. In comparison to the only existing study on PTSD-related hospitalizations in the United States, there were 10 times fewer hospitalizations in France over a similar 10-year span, despite the US population being approximately five times larger [19]. Additionally, a study in Northern Ireland reported over 37,000 PTSD-related hospitalizations in 2008 alone [30], nearly double the number of PTSD hospitalizations in France in 2022, the year with the highest number of hospitalizations. This discrepancy may be partly explained by variations in PTSD prevalence between countries, with Northern Ireland and the US experiencing particularly high rates [11, 30].

Furthermore, while two-thirds of hospitalizations with PTSD occurred in psychiatric facilities in our study (59.8%), the remaining cases were observed in general medical services, where the prevalence of non-psychiatric comorbidities was significantly higher (77% vs. 17% in psychiatric settings). This finding aligns with existing scientific literature, which indicates that individuals with PTSD are frequently hospitalized in non-psychiatric services [7, 16]. This is likely due to dysregulation of the stress system, which increases the risk of cardiovascular diseases, diabetes, and inflammation [7], and physical consequences of the trauma.

Over the 10-year period, the number of hospitalizations with PTSD increased in both psychiatric and general hospitals across France. A similar increase in PTSD-related hospitalizations was reported in the US in the early 2000s [19], while in Korea, a study from 2011 to 2017, observed an increase in the incidence of PTSD in the general population [31]. While our study covers a more recent period, these findings are consistent with broader trends observed in the literature. This rise can be attributed to several social, political, and health system factors.

France has faced a series of traumatic events, including terrorist attacks, natural disasters, and more recently, the COVID-19 pandemic [32]. These events have likely contributed to the rising prevalence of PTSD by increasing the population’s exposure to trauma. In particular, scientific literature reported an increase in PTSD cases after the COVID-19 pandemic [23, 25]. Supporting this, we observed in our results a more significant increase in the inpatient prevalence of PTSD in psychiatric facilities after 2019. Additionally, the COVID-19 pandemic may have specifically delayed interventions for PTSD due to restricted access to outpatient care and other mental health services. This lack of timely intervention could have worsened PTSD symptoms, increasing the severity of the disorder and ultimately leading to a higher likelihood of hospitalization for affected individuals.

Moreover, changes in diagnostic practices and an increased awareness of PTSD among healthcare providers could also explain part of this trend. As the understanding of PTSD has evolved, it is possible that more accurate diagnoses and improved coding practices in hospital settings have contributed to the observed increase in PTSD-related hospitalizations [5, 9, 33–35]. Specific initiatives aimed at improving the recognition and treatment of trauma-related disorders, including PTSD, such as the creation of the National Centre for Resources and Resilience (Cn2r) in 2019 in France, have helped raised awareness both among the general public and healthcare professionals [36]. As a result, this may have led to higher detection rates and more frequent hospitalizations for severe cases, reflecting an improved identification of the disorder.

These contextual factors, combined with delays in access to care due to the healthcare system’s response to various crises, suggest that the rise in hospitalizations could thus reflect both an increase in the actual prevalence of PTSD and changes in healthcare practices [32]. Future research should aim to disentangle the relative contributions of these factors to better understand the drivers behind this trend.

Additionally, there was a notable decline in the average age of patients hospitalized with PTSD, accompanied by an increase in the number of part-time hospitalizations in psychiatric facilities. The decrease in the median age of individuals can be explained by the fact that the disorder is increasingly common among adolescents, particularly since the pandemic crisis [25, 37]. These trends may also indicate a possible earlier detection and prompter initiation of care for patients, particularly over the past two decades with the implementation of effective treatments such as cognitive behavioral therapy and Eye Movement Desensitization and Reprocessing (EMDR) [38]. Moreover, the increase in part-time hospitalizations follows international recommendations aimed at reducing the length of stay and developing alternatives to full-time hospitalizations [39, 40].

The comparison of hospitalizations by sex revealed notable differences: females were more often hospitalized, were younger, and had longer hospital stays compared to males in psychiatric services. This aligns with previous studies indicating a higher prevalence of PTSD among women in the general population, as well as in primary care settings, which may explain the higher proportion of female hospitalizations [11, 16, 31, 41–43]. Research indicates that women have higher rates of PTSD due to greater exposure to trauma, such as sexual abuse, as well as a heightened vulnerability to developing the disorder [11]. Even when accounting for trauma exposure, women still show a greater risk, suggesting that both environmental and biological factors contribute to this difference. Women also appear to experience more severe PTSD [42, 43], which could explain the longer duration of hospitalization in psychiatric facilities. Similarly, between 2002 and 2013, women aged 20 to 44 exhibited the highest rates of hospitalization in the USA and tended to have longer hospital stays [19]. This trend of young women being hospitalized therefore appears to persist over time.

Prevalent comorbidities observed in psychiatric settings included mood disorders, stress-related disorders (including anxiety disorders), and alcohol-related disorders. Previous literature similarly identifies these disorders as frequently co-occurring with PTSD [6, 44]. We found that two-thirds of individuals hospitalized in psychiatric facilities with a PTSD diagnosis had a comorbid disorder. While some studies, such as Bryant’s, report higher comorbidity rates of up to 91%, others indicate rates closer to 50%, highlighting variability in the literature [44–46]. Given that our study focuses on a hospitalized population, which is typically more severe, our observed rate fits within the expected ranges for this group. In general hospital wards, nonspecific diagnoses (Z5: problems related to economic and social circumstances; R5: symptoms and signs involving the digestive system and abdomen) were the most frequently associated with PTSD hospitalizations, accounting for 15.4% of hospitalizations. These related diagnoses reflect on one hand, the socioeconomic distress faced by patients suffering from PTSD [31] and, on the other hand, the more general physical symptoms that may lead them to seek medical care [18]. Some studies have suggested that economic disadvantage may be a risk factor for developing PTSD [31, 47]. Alcohol-related disorders, mood disorders, and stress-related disorders were subsequently the most frequent, but in proportions much lower than in psychiatric facilities, and a very small percentage of patients had no comorbidities.

### Strengths and limitations

We present here the first nationwide study in France to examine hospitalizations with a diagnosis of PTSD. We conducted comparisons based on sex and examined the evolution of patient characteristics and hospitalizations over the past decade. However, there are limitations. First we focused solely on hospitalizations with a primary or secondary diagnosis code for PTSD. It is likely that the prevalence of PTSD in hospitalizations is underestimated in our study, as not all patients disclose their PTSD symptoms, leading to underdiagnosis [29]. Research suggests that individuals with PTSD often seek medical treatment for physical symptoms from primary care physicians without disclosing their psychiatric symptoms or trauma history [28]. Additionally, this study is based on data from the French National Hospitals Database (PMSI), which records only hospitalizations. Consequently, cases of PTSD treated in outpatient settings, where most patients receive care, are not included. Thus, the results are representative only of hospitalized PTSD patients and may not be generalizable to all individuals with PTSD in France. Moreover, the PMSI system does not necessarily rely on standardized diagnoses, as they may be based on varying criteria or questionnaires and can be made by specialists other than psychiatrists. This limitation should be considered when interpreting our findings. Furthermore, this descriptive study examined factors such as age, length of hospitalization, and other relevant variables like sex, and their evolution between 2013 and 2022. It was not intended to study the impact of potential confounders such as demographic variables or comorbid conditions, nor to assess how results might vary when considering different age groups or comorbidities. Future studies should be specifically designed to evaluate confounders and use sensitivity analyses to refine and validate the findings. Finally, we did not assess suicidal behavior in our study, despite it being a common comorbidity of PTSD [48].

Our results highlight the growing demand for mental health services related to PTSD, which calls for strategic interventions at the policy level. One key area for improvement is the strengthening of outpatient services. Ensuring timely access to outpatient mental health care, particularly for trauma survivors, could help prevent the worsening of PTSD symptoms and reduce the need for hospitalization. Initiatives such as expanding trauma-focused therapy options and increasing the availability of specialized PTSD care in ambulatory settings would be crucial in addressing this issue. France has already made efforts to improve post-trauma care with the creation of regional ambulatory services specializing in psychotraumatology in 2019 [36]. Our findings suggest that further investment in these services is necessary to keep up with the increasing demand. Expanding these centers and improving accessibility, especially in underserved areas, could help alleviate the burden on hospital systems. Additionally, further integrating mental health care into primary care settings is another important lever for action. Training general practitioners to better recognize PTSD symptoms could facilitate earlier interventions, which in turn may help prevent severe cases from escalating to the point of requiring hospitalization. At the clinical level, improving early detection and ensuring ongoing follow-up for PTSD patients is essential. Clinicians should be encouraged to use standardized PTSD assessment tools in both outpatient and inpatient settings to ensure more consistent diagnoses. This approach could help optimize treatment pathways and reduce the progression of symptoms that lead to hospitalizations.

In conclusion, our study is one of the first national studies to assess hospitalizations with a diagnosis of PTSD, providing detailed insights and evaluation of hospitalizations over a period of 10 years. We observed a significant increase in PTSD-related hospitalizations, with a more pronounced rise in psychiatric facilities, particularly after the COVID-19 pandemic. Females were younger than males in both facilities and tended to have longer stays in psychiatric facilities. Over time, the median age of individuals with PTSD has decreased, and the number of part-time hospitalizations has increased. The distribution of hospitalizations across psychiatric and general services highlights the complex nature of PTSD and its association with various comorbidities, necessitating comprehensive healthcare approaches for effective management. Further investigations are needed to better understand the factors contributing to hospitalizations across different healthcare settings, with particular attention to optimizing care strategies for younger individuals. Future research should also focus on examining trends in outpatient care and evaluating potential interventions, such as early detection and improved access to mental health services. Implementing these strategies could help mitigate the growing number of PTSD-related hospitalizations, ultimately reducing the burden on healthcare systems and improving patient outcomes.

## Data Availability

The data that support the findings of this study are available upon request from the corresponding author, FD. The data are not publicly available owing to restrictions related to privacy concerns.
